# Prevalence and factors associated with post-traumatic stress disorder in healthcare workers exposed to COVID-19 in Wuhan, China: a cross-sectional survey

**DOI:** 10.1186/s12888-021-03589-1

**Published:** 2021-11-15

**Authors:** Lingling Pan, Qiancheng Xu, Xia Kuang, Xiancui Zhang, Fengxia Fang, Liling Gui, Mei Li, Boris Tefsen, Lei Zha, Huan Liu

**Affiliations:** 1 Department of Cardiology, The First Affiliated Hospital of Wannan Medical College, Wuhu, 241000 Anhui China; 2 Department of Critical Care Medicine, Zhongnan Hospital of Wuhan University, Wuhan, 430071 Hubei China; 3 Department of Critical Care Medicine, The First Affiliated Hospital of Wannan Medical College, Wuhu, 241000 Anhui China; 4 Nursing Department, The First Affiliated Hospital of Wannan Medical College, No.2, West Road of Zheshan, Wuhu, 241000 Anhui China; 5 Department of Radiation and Medical Oncology, Zhongnan Hospital of Wuhan University, Wuhan, 430071 Hubei China; 6 Department of Intensive Care Unit, The Central Hospital of Wuhan Tongji Medical College, Huazhong University of Science and Technology, Wuhan, 430074 Hubei China; 7 Ronin Institute, Montclair, NJ 07043 USA; 8 Emergency and Critical Care Unit, Conch Hospital of Anhui Medical University, No. 327, Jiuhua Road, Wuhu, 241000 Anhui China; 9 Department of Biological Science, Xi’an Jiaotong-Liverpool University, Suzhou, 215123 Jiangsu China

**Keywords:** COVID-19, PTSD, Mental health, Healthcare workers, Risk factors

## Abstract

**Background:**

The COVID-19 pandemic has posed significant threats to both the physical and psychological health of healthcare workers working in the front-line combating COVID-19. However, studies regarding the medium to long term impact of COVID-19 on mental health among healthcare workers are limited. Therefore, we conducted this cross-sectional survey to investigate the prevalence, factors and impact of post-traumatic stress disorder (PTSD) in healthcare workers exposed to COVID-19 8 months after the end of the outbreak in Wuhan, China.

**Methods:**

A web-based questionnaire was delivered as a link via the communication application WeChat to those healthcare workers who worked at several COVID-19 units during the outbreak (from December 2019 to April 2020) in Wuhan, China. The questionnaire included questions on social-demographic data, the post-traumatic stress disorder checklist-5 (PCL-5), the family care index questionnaire (Adaptation, Partnership, Growth, Affection and Resolve, APGAR), and the quality-of-life scale (QOL). The prevalence, risk and protective factors, and impact of PTSD on healthcare workers were subsequently analyzed.

**Results:**

Among the 659 participants, 90 healthcare workers were still suffering from PTSD 8 months after the end of the outbreak of COVID-19 in Wuhan, in which avoidance and negative impact were the most affected dimensions. Suffering from chronic disease, experiencing social isolation, and job dissatisfaction came up as independent risk factors for PTSD, while obtaining COVID-19 related information at an appropriate frequency, good family function, and working in well-prepared mobile cabin hospitals served as protective factors. The impact of PTSD on COVID-19 exposed healthcare workers was apparent by shortened sleeping time, feeling of loneliness, poorer quality of life and intention to resign.

**Conclusions:**

Eight months after the end of the COVID-19 outbreak in Wuhan, the level of PTSD in healthcare workers exposed to COVID-19 was still high. Apart from the commonly recognized risk factors, comorbid chronic disease was identified as a new independent risk factor for developing PTSD. For countries where the pandemic is still ongoing or in case of future outbreaks of new communicable diseases, this study may contribute to preventing cases of PTSD in healthcare workers exposed to infectious diseases under such circumstances.

## Background

Coronavirus disease 2019 (COVID-19) is a highly contagious acute infectious respiratory disease caused by severe acute respiratory syndrome coronavirus-2 (SARS-CoV-2) [1]. The outbreak of COVID-19 emerged in Wuhan (China) in the last month of 2019 and the subsequent spread outside of China led the World Health Organization (WHO) Emergency Committee to declare a Public Health Emergency of International Concern (PHEIC) on January 30, 2020, and a pandemic on March 11, 2020 [2]. The COVID-19 pandemic not only causes physical diseases, but also leads to great psychological distress, resulting in high level of anxiety, depression, burnout syndrome and PTSD [3–5]. An important group experiencing severe distress consists of the doctors and nurses caring for COVID-19 patients, which is not surprising as several studies have reported that medical staff comprises one of the most vulnerable sectors in the population during global events [6–8]. As of the writing (May 14, 2021), with more than 162 million detected infections worldwide and the pandemic far from being contained, investigating the psychological impact of this pandemic on healthcare workers (HCWs) has become increasingly important, especially the long-lasting impact on those working at the early stage of the pandemic. Post-traumatic stress disorder (PTSD) refers to an individual’s delayed appearance and long-lasting mental disorder caused by sudden, catastrophic or threatening life events [9, 10]. It is an emotional disorder dominated by fear and anxiety, which might lead to depression, helplessness, memory impairment, and decreased work quality [11]. Being isolated, working in high-risk positions, being nurses, and having contact with infected people are known risk factors for PTSD [3, 12]. The COVID-19 pandemic very likely also exerts a similar impact on HCWs. Indeed, several studies have reported that a proportion of first-line HCWs developed PTSD and that their work and life were affected significantly [13–15]. A quantitative study indicated that the outbreak of COVID-19 in Wuhan had a clear impact on the mental health of local HCWs, with 22.4% reporting moderate and 6.2% severe disturbances [7]. Other studies have also investigated the PTSD status and risk factors of front-line HCWs [16–18]. However, most of these studies investigated short-term PTSD symptoms soon after the outbreak, and any evidence of long-term PTSD related to COVID-19 is limited thus far. Therefore, in the present study, we aim to investigate the prevalence of PTSD and corresponding factors in HCWs exposed to COVID-19 8 months after the end of the outbreak in Wuhan.

## Methods

### Study design

A web-based questionnaire was delivered between November and December 2020 as a link via a communication application (WeChat) to those HCWs, who worked at several COVID-19 units (including the emergency department, outpatient, fever clinic, intensive care unit, infection ward, isolation ward, mobile cabin hospital) in Wuhan, from the onset of the COVID-19 outbreak. In China, HCWs are not used to email for their daily job, instead, the instant messenger WeChat is widely used to discuss work and transfer files. Therefore, in the present study we used WeChat to deliver the link of the questionnaire for the purpose of convenience, as it is a quick, easy way to reach the target population and most likely to yield a response. As WeChat only allows one account per person, verified by identification card, this platform is very suitable to gather unique anonymized responses, yet without the chance of having a single person submitting multiple questionnaires. In order to exclusively receive completed questionnaires, the online version of the questionnaire in WeChat could only be submitted if all required questions had been answered. The study was approved by the Ethics Committee of Conch Hospital of Anhui Medical University (approval number 20201025). All participants joined the survey voluntarily and provided written informed consent before answering the questionnaire. All data for the study were collected and analyzed anonymously.

### Participants

HCWs having cared for COVID-19 patients in Wuhan were recruited by snowball sampling [19]. Inclusion criteria were HCWs who worked at a COVID-19 designated institution, had direct contact with COVID-19 patients, or had close contact with those provided health care service to COVID-19 patients. The post-hoc analysis indicated that all participants had worked in the designated institutions for more than 7 days; hence we did not set an exclusion criterion to a minimal working time in the designated institutions. Exclusion criteria were non-HCWs, and provision of invalid answers to the questionnaire.

### Instruments

#### Social-demographic, job and COVID-19 related variables

Social-demographic variables in the questionnaire include age, gender, marital status, education level (college diploma, bachelor, or master and above), occupation (doctor, nurse or non-clinical; which includes nurses and doctors carrying out administrative and logistics tasks during the outbreak of COVID-19 due to lack of human resource in these departments), whether suffering from chronic diseases, and whether being the only child. Job-related variables include work experience (< 2 years, 2–5 years, 6–10 years, 11–20 years and > 20 years), position level (unrated, elementary, intermediate, advanced) and department (emergency department, outpatient, fever clinic, intensive care unit, infection ward, isolation ward, mobile cabin hospital). COVID-19 related variables include the number of working days during the epidemic, experience in caring for patients suffering from other respiratory infectious diseases (e.g. SARS, MERS, influenza), direct care for COVID-19 patients, occupational exposure without protection, getting infected, relatives or close friends getting infected or died of COVID-19 (diagnosed by qPCR or clinical diagnostic criteria), having been in quarantine and time of quarantine due to COVID-19, the experience of social isolation, received psychological assistance, having received an award for fighting COVID-19, frequency of obtaining COVID-19 related information (rarely, sometimes, always), sleeping time (< 5 h, 6–8 h, > 8 h), job satisfaction (dissatisfied, neutral, satisfied), intention to resign, feeling of loneliness (rarely, sometimes, often).

#### PCL-5, family care index questionnaire, and quality of life scale

The presence and severity of PTSD symptoms were measured by the post-traumatic stress disorder checklist-5 (PCL-5), containing 20 self-report items, with high reliability and validity [20, 21]. Each item of PCL-5 has five-point Likert scare (0 = not at all, 1 = a little bit, 2 = moderately, 3 = quite a bit and 4 = extremely). Individual’s score of PCL-5 ranges from 0 to 80, and the cut-off point score for diagnosis PTSD was set as more than or equal to 33, with a higher score indicating more serious PTSD symptoms [22].

The Family function was assessed with the family care index questionnaire (Adaptation, Partnership, Growth, Affection and Resolve, APGAR) designed by the University of Washington [23]. The questionnaire contains five dimensions: adaptation, partnership, growth, affection and resolve. The questionnaire uses a Likert 3-level scoring response model, 0 point (almost never), 1 point (sometimes), 2 points (often like this). The full score of the scale is 10 points, and 0 to 3 points indicates a severe family dysfunction, 4 to 6 points indicates a moderately impaired family function, and a score of 7 to 10 indicates a good family function.

Quality of life was measured with the Chinese QOL questionnaire [24], with a total of 6 items, assessing the physical health, mental health, economic status, work or study status, family relationship and non-family peers’ relationship, respectively. The answers are divided into 5 levels (1 = very poor, 2 = poor, 3 = fair, 4 = good, and 5 = very good).

### Statistical analysis

The sample size was calculated at a 5% margin of error, 95% of sampling confidence level in the estimated population size of 150,000, indicating 384 participants were required in the study, based on methods described in the review [25]. Continuous variables were summarized as either means and standard deviations or medians and interquartile range as appropriate. Categorical variables were described as frequencies and percentages. The differences between groups with or without PTSD were analyzed by Fisher’s exact test and the Mann – Whitney *U* test for continuous variables. Factors associated with PTSD were identified using logistic regression. Adjustment for the following variables (determined a priori) was conducted in a forward stepwise manner: age, suffered from chronic diseases, being an only child, occupational exposure without protection, relatives or friends being infected, relatives or friends died of COVID-19, the experience of social isolation, job satisfaction, frequency of obtaining COVID-19 related information, working department, and variables with *p* ≤ 0.2 in the univariable analysis. Odds ratios (OR) and 95% confidence intervals (CI) were reported. Two-tailed *p* <  0.05 was considered statistically significant. All analyses were performed using R software version 3.6.2 (R Foundation for Statistical Computing).

## Results

### Participant’s characteristics

A total of 667 HCWs participated in the survey, and after the exclusion of 8 questionnaires because of invalid answers, 659 participants were included in the final analysis. Among the participants, 573 (86.9%) were nurses, 55 (8.3%) were doctors, and 31 (4.7%) were non-clinical HCWs. Females accounted for 90.6% (597) of the participants. Most of the HCWs engaged in fighting COVID-19 were relatively young, 223 (33.8%) aged between 31 to 40 years, and 316 (48%) were younger than 30 years. The majority of participants in this survey had a bachelor degree (509, 77.2%) and had a primary (377, 57.2%) to middle (221, 33.5%) job title. 425 (64.5%) participants were married, while 234 (35.5%) were unmarried/divorced/widowed (Table 1).

**Table 1:** Characteristics of healthcare workers exposed to COVID-19

Characteristics		All HCWs	Non-PTSD	PTSD	*p*
			PCL-5 < 33	PCL-5 ≥ 33	
		659	569	90	
Gender (%)	Male	62 (9.4)	57 (10.0)	5 (5.6)	0.249
	Female	597 (90.6)	512 (90.0)	85 (94.4)	
Age (%)	18–30	316 (48.0)	279 (49.0)	37 (41.1)	0.19
	31–40	223 (33.8)	185 (32.5)	38 (42.2)	
	> 41	120 (18.2)	105 (18.5)	15 (16.7)	
Education level (%)	College diploma	80 (12.1)	69 (12.1)	11 (12.2)	0.979
	Bachelor	509 (77.2)	439 (77.2)	70 (77.8)	
	Master and above	70 (10.6)	61 (10.7)	9 (10.0)	
Marital status (%)	Unmarried	213 (32.3)	186 (32.7)	27 (30.0)	0.388
	Married	425 (64.5)	363 (63.8)	62 (68.9)	
	Divorced/widowed	21 (3.2)	20 (3.5)	1 (1.1)	
Occupation (%)	Doctor	55 (8.3)	50 (8.8)	5 (5.6)	0.263
	Nurse	573 (86.9)	490 (86.1)	83 (92.2)	
	Non-clinical	31 (4.7)	29 (5.1)	2 (2.2)	
Position level (%)	Unrated	34 (5.2)	29 (5.1)	5 (5.6)	0.653
	Elementary	377 (57.2)	331 (58.2)	46 (51.1)	
	Intermediate	221 (33.5)	186 (32.7)	35 (38.9)	
	Advanced	27 (4.1)	23 (4.0)	4 (4.4)	
Suffered from chronic diseases (%)	No	580 (88.0)	510 (89.6)	70 (77.8)	0.002
	Yes	79 (12.0)	59 (10.4)	20 (22.2)	
The only child (%)	No	466 (70.7)	408 (71.7)	58 (64.4)	0.2
	Yes	193 (29.3)	161 (28.3)	32 (35.6)	
Time of work during the outbreak (median [IQR])		30 [15–60]	30 [15–60]	30 [15.25–60]	0.752
Experience in fighting other infectious diseases (%)	No	601 (91.2)	519 (91.2)	82 (91.1)	1
	Yes	58 (8.8)	50 (8.8)	8 (8.9)	
Direct care for COVID-19 patients (%)	No	174 (26.4)	151 (26.5)	23 (25.6)	0.946
	Yes	485 (73.6)	418 (73.5)	67 (74.4)	
Occupational exposure without protection (%)	No	580 (88.0)	508 (89.3)	72 (80.0)	0.019
	Yes	79 (12.0)	61 (10.7)	18 (20.0)	
Be infected (%)	No	633 (96.1)	547 (96.1)	86 (95.6)	1
	Yes	26 (3.9)	22 (3.9)	4 (4.4)	
Relatives or friends be infected (%)	No	235 (35.7)	210 (36.9)	25 (27.8)	0.118
	Yes	424 (64.3)	359 (63.1)	65 (72.2)	
Relatives or friends died of COVID-19 (%)	No	506 (76.8)	448 (78.7)	58 (64.4)	0.004
	Yes	153 (23.2)	121 (21.3)	32 (35.6)	
Experienced quarantine (%)	No	340 (51.6)	296 (52.0)	44 (48.9)	0.661
	Yes	319 (48.4)	273 (48.0)	46 (51.1)	
Time of quarantine (median [IQR])		3 [3–14]	3 [3–14]	6 [3–14]	0.436
Experienced social isolation (%)	No	512 (77.7)	458 (80.5)	54 (60.0)	< 0.001
	Yes	147 (22.3)	111 (19.5)	36 (40.0)	
Received psychological assistance (%)	No	563 (85.4)	489 (85.9)	74 (82.2)	0.442
	Yes	96 (14.6)	80 (14.1)	16 (17.8)	
Awarded (%)	No	438 (66.5)	371 (65.2)	67 (74.4)	0.108
	Yes	221 (33.5)	198 (34.8)	23 (25.6)	
Frequency of obtaining COVID-19 related information (%)	Rarely	164 (24.9)	133 (23.4)	31 (34.4)	0.057
	Sometimes	235 (35.7)	210 (36.9)	25 (27.8)	
	Always	260 (39.5)	226 (39.7)	34 (37.8)	
Job satisfaction (%)	Dissatisfied	111 (16.8)	79 (13.9)	32 (35.6)	< 0.001
	Average	353 (53.6)	304 (53.4)	49 (54.4)	
	Satisfied	195 (29.6)	186 (32.7)	9 (10.0)	
Department (%)	Outpatient /Emergency	101 (15.3)	80 (14.1)	21 (23.3)	0.027
	ICU	48 (7.3)	37 (6.5)	11 (12.2)	
	Infection ward	100 (15.2)	86 (15.1)	14 (15.6)	
	Isolation ward	155 (23.5)	137 (24.1)	18 (20.0)	
	Mobile cabin hospital	255 (38.7)	229 (40.2)	26 (28.9)	
Work experience (median [IQR])		8 [4–15]	8 [4–15]	9 [5–15]	0.555
APGAR (median [IQR])		8 [5–10]	8 [5–10]	5 [4–8]	< 0.001

In this survey, there were 90 (13.7%) HCWs with a PCL-5 score ≥ 33 and were then considered as suffering from PTSD. Compared with those participants without PTSD, the incidence rate of suffering from chronic diseases (20, 22.2% vs. 59, 10.4%, *p* = 0.002), occupational exposure without protection (18, 20% vs. 61, 10.7%, *p* = 0.019), relatives or close friends died of COVID-19 (32, 35.6% vs. 121, 21.3%, *p* = 0.004), experience of social isolation (36, 40% vs. 111, 19.5%, *p* <  0.001), and dissatisfaction with job (32, 35.6% vs. 79, 13.9%, *p* <  0.001) were higher in HCWs with PTSD. By contrast, there was no statistical significance in the rate of the following: having had the experience in caring other infectious diseases, having been infected, relatives or close friends gotten infected, experienced social isolation, received psychological assistance, awarded for fighting COVID-19, as well as the time of working during the epidemic, time of quarantine due to COVID-19 and frequency of obtaining COVID-19 related information. HCWs working in the so-called mobile cabin hospitals (basic hospitals setup in gymnasiums and conference centers in March 2020, mostly used to treat patients with COVID-19 who had mild symptoms [26]) had the lowest rate of PTSD (26, 10.2%). Moreover, the APGAR score of family function was lower in the PTSD group compared with non-PTSD participants (5, 4–8, vs. 8, 5–10, *p* <  0.001) (Table 1).

### Dimension’s characteristics of PTSD in PCL-5

The score of each dimension was shown in Table 2. Compared with non-PTSD HCWs, those with PTSD had a higher score in all six dimensions, among which avoidance (0.00 [0.00, 1.00] vs 2.00 [1.00, 2.00], *p* <  0.001) and negative impact (0.75 [0.25, 1.00] vs 2.00 [1.25, 2.00], *p* <  0.001) were the most affected dimensions.

**Table 2:** Scores of each dimension of PTSD (median [IQR])

Dimensions	Scores	Non-PTSD	PTSD	P
		PCL-5 < 33	PCL-5 ≥ 33	
Intrusion	0.80 [0.40, 1.20]	0.80 [0.40, 1.20]	1.60 [1.40, 2.00]	< 0.001
Avoidance	0.50 [0.00, 1.00]	0.00 [0.00, 1.00]	2.00 [1.00, 2.00]	< 0.001
Negative affect	1.00 [0.25, 1.25]	0.75 [0.25, 1.00]	2.00 [1.25, 2.00]	< 0.001
Anhedonia	1.00 [0.00, 1.67]	1.00 [0.00, 1.33]	2.00 [2.00, 2.67]	< 0.001
Dysphoric arousal	1.00 [0.25, 1.50]	1.00 [0.00, 1.25]	2.00 [1.75, 2.50]	< 0.001
Anxious arousal	1.00 [0.00, 2.00]	1.00 [0.00, 1.00]	2.00 [2.00, 2.50]	< 0.001
PCL-5 total	18.00 [8.00, 26.00]	15.00 [7.00, 22.00]	37.00 [35.00, 41.00]	< 0.001

### Impact of PTSD on participants

Compared with non-PTSD HCWs, PTSD participants reported a higher score of panic (5.00 [5.00, 7.00] vs. 4.00 [2.00, 5.00], *p* <  0.001), shorter sleeping time, poorer quality of life (18.00 [16.00, 19.00] vs. 20.00 [18.00, 23.00], *p* <  0.001), and more frequent feelings of loneliness. Moreover, 61.1% (55) of HCWs with PTSD intended to resign from their current job, compared to 32% (182) in those without PTSD. Only a small proportion (39, 6.9%) of participants without PTSD thought they needed to seek psychological assistance, while 44.4% (40/90) of the participants with PTSD were planning to consult with a psychologist (Table 3).

**Table 3:** The impact of PTSD on healthcare workers

Characteristics		All HCWs	Non- PTSD	PTSD	*p*
			PCL-5 < 33	PCL-5 ≥ 33	
Seek psychological assistance (%)	Need	580 (88.0)	530 (93.1)	50 (55.6)	< 0.001
	No need	79 (12.0)	39 (6.9)	40 (44.4)	
Sleeping time (%)	< 5 h	56 (8.5)	41 (7.2)	15 (16.7)	0.007
	6–8 h	544 (82.5)	474 (83.3)	70 (77.8)	
	> 8 h	59 (9.0)	54 (9.5)	5 (5.6)	
Frequency of feeling of loneliness (%)	Rarely	348 (52.8)	332 (58.3)	16 (17.8)	< 0.001
	Sometimes	245 (37.2)	201 (35.3)	44 (48.9)	
	Often	66 (10.0)	36 (6.3)	30 (33.3)	
Intention to resign (%)	No	422 (64.0)	387 (68.0)	35 (38.9)	< 0.001
	Yes	237 (36.0)	182 (32.0)	55 (61.1)	
Panic (median [IQR])		4 [2–5]	4 [2–5]	5 [5–7]	< 0.001
Quality of life (median [IQR])		20 [18–22]	20 [18–23]	18 [16–19]	< 0.001

### Risk factors associated with PTSD in HCWs

In order to identify factors that are associated with PTSD, we have run a logistic regression model. The results indicate as independent risk factors for PTSD: suffering from chronic diseases (2.19, 1.11–4.24, *p* = 0.02), experienced social isolation (2.38, 1.37–4.13, *p* = 0.002), and dissatisfaction with job (4.92, 1.30–6.23, *p* = 0.012), while obtaining COVID-19 related information at an appropriate frequency (sometimes, (0.51, 0.26–0.98, *p* = 0.04)), good function of family (0.27, 0.16–0.46, *p* <  0.001), and working in the well-prepared mobile cabin hospitals (0.46, 0.23–0.94, *p* = 0.03) were protective factors of developing PTSD (Table 4).

**Table 4:** Factors associated with PTSD

Predictive factors		OR	95% CI	P
Suffering from chronic diseases	Yes	2.19	1.11–4.24	0.02
Experience of social isolation	Yes	2.38	1.37–4.13	0.002
Frequency of obtaining COVID-19 related information	Sometimes	0.51	0.26–0.98	0.04
Department	Mobile cabin hospital	0.46	0.23–0.94	0.03
APGAR	Good function of family	0.27	0.16–0.46	< 0.001
Job Satisfaction	Dissatisfied	4.92	1.30–6.23	0.012

## Discussion

The prevalence of PTSD in this study is 13.7% (90/659) which is lower than that reported in a recent study (61.8%) [27]. In that study, only front-line nurses were included and were all screened with PTSD immediately after having worked in COVID-19 units. Other studies have indicated that nurses were more vulnerable than other HCWs to PTSD [16, 28]. In our analysis, we also included other HCWs besides nurses; therefore, the impact of COVID-19 on HCWS can be more fully evaluated, although nurses still accounted for the majority of the participants (86.9%, 573/659). Furthermore, the time points of the PTSD screening were quite different. In the present study, all participants were screened 8 months after the exposure to COVID-19. The results of those previous studies, together with the results reported here, suggest that the incidence of COVID-19-related PTSD decreases over time. A similar decreasing trend was also reported in PTSD caused by a devastating earthquake, in which the PTSD rate was 8.4% 1 month after the earthquake, while decreasing to 4.3% 3 months later, and then dropping to 3.4% half a year later [29].

In terms of factors associated with PTSD, we identified that participants with a poorer score of family function, experienced social isolation and dissatisfaction with their job were at higher risk of developing PTSD, which highlighting the importance of support from supervisors, colleagues, family and society. This is in line with previous studies, as these factors were also frequently reported in HCWs exposed to SARS [30–32]. In the present study, working in mobile cabin hospitals was identified as an independent protective factor of PTSD. Compared with other hospital departments, mobile cabin hospitals in China were well prepared with enough protective equipment, staff and well-organized infection prevention and control strategies, which increased the sense of security and hence decreased the extent of the impact of COVID-19 itself. Likewise, other studies have reported that a structured unit with perceived safety of the working environment enhanced the resilience against PTSD [30, 33]. Therefore, it is recommended to conduct workplace risk assessment and occupational health surveillance to establish the most effective preventive and protective measures in hospitals to eliminate risks of infection of COVID-19 among HCWs [34]. Interestingly, in our study, we found that chronic physical disease was associated with developing PTSD in HCWs, something that has not been reported before to our knowledge. As medical professionals, HCWs know that chronic diseases are independent risk factors for developing severe disease and would cause higher mortality in COVID-19. Therefore, this professional knowledge might cause the fear of developing the serious disease when infected with COVID-19 and subsequently cause higher stress than those without physical chronic diseases. Previous research has reported that psychiatric disorders are risk factors for developing PTSD in SARS and MERS [35, 36]. Therefore, both from a physical, as well as a psychological point of view, it is recommended to avoid deploying HCWs with chronic diseases to care for COVID-19 patients or other communicable diseases.

Regarding the impact of PTSD on daily life, although the frequency of panic did not show any statistically significant difference between participants with or without PTSD, those with PTSD did have a poorer score of quality of life, often feel lonely, and sleep less than that without PTSD. Strikingly, 44.4% of the participants with PTSD thought they were in need of psychological assistance. A study done immediately after the outbreak was brought under control in Wuhan demonstrated that of all HCWs caring for COVID-19 patients included, 36.3% had accessed psychological materials, 50.4% had accessed psychological resources available through media, and 17.5% had participated in counselling or psychotherapy [7]. As it was demonstrated that the workplace is a suitable arena for implementing mental health interventions to promote psychological resources and resilience and provide social and emotional support to HCWs [37], we recommend hospitals to allocate more resources to psychological assistance for staff that were directly or indirectly exposed to COVID-19. Moreover, the intention to resign in participants with PTSD was twice higher than those without PTSD in the present study, which is significantly consistent with the dimension of avoidance score. Combined with this evidence, it seems good if hospitals would offer opportunities to HCWs at risk for PTSD (or all, depending on capacity) to rotate to other positions for certain periods, to lower the prolonged fear related to caring for COVID-19 patients, as that was also suggested in a study on work-related PTSD [38].

### Strength and limitations

The strength of this research is the medium to long term information of the mental health of HCWs 8 months after the end of the COVID-19 pandemic in Wuhan, China. Our research provides evidence to design strategies to prevent PTSD among HCWs during the COVID-19 pandemic. However, there are some limitations in this study. First of all, this study only investigated medical staff in a few local hospitals in Wuhan and might not be generalizable to all medical staff. Second, the questionnaire used in the survey was not an adapted version specified for COVID-19 related PTSD, which might influence its reliability and validity. Third, the prevalence of PTSD in the present study was identified by a self-reported questionnaire which is a screening tool rather than a diagnostic tool, therefore it might overestimate the incidence of PTSD. Clinical diagnosis of PTSD should be based on a comprehensive clinical evaluation performed by an experienced psychiatrist thus ruling out other conditions that could also cause similar symptoms. Moreover, participants in the survey all joined voluntarily, those HCWs with PTSD, especially those with severe symptoms, might be reluctant to participate in the study and subsequently lead to under-reporting. Finally, the prevalence and factors associated with PTSD may change dynamically as the pandemic is ongoing; thus, evidence generated by the present study might not be suitable for other periods and or regions. Therefore, a multi-center survey with multiple time points and a large sample size is still warranted to assess the PTSD level of HCWs who exposed to COVID-19 patients.

## Conclusion

The prevalence of PTSD was 13.7% among HCWs 8 months after the exposure of COVID-19. Suffering from chronic disease, the experience of social isolation, dissatisfied with the job were independent risk factors for PTSD, while obtaining COVID-19 related information at an appropriate frequency, good function of family, and working in the well-prepared mobile cabin hospitals were protective factors of developing PTSD. The impact of PTSD on HCWs was shorter sleeping time, feeling of loneliness, poorer quality of life and intention to resign.

## Data Availability

The datasets used and/or analyzed during the current study are available from the corresponding author (L.Z or H. L) on reasonable request.

## Abbreviations

COVID-19: Coronavirus disease 2019
SARS-CoV-2: Severe acute respiratory syndrome coronavirus-2
PTSD: Post-traumatic stress disorder
HCWs: Healthcare workers
SARS: Severe acute respiratory syndrome
MERS: Middle East respiratory syndrome
PCL-5: Post-traumatic stress disorder checklist-5
APGAR: Adaptation, partnership, growth, affection and resolve
